# LncRNA HCP5 knockdown inhibits high glucose-induced excessive proliferation, fibrosis and inflammation of human glomerular mesangial cells by regulating the miR-93-5p/HMGA2 axis

**DOI:** 10.1186/s12902-021-00781-y

**Published:** 2021-06-29

**Authors:** Xuan Wang, Yan Liu, Jian Rong, Kai Wang

**Affiliations:** 1 Department of International Medical Center, Tianjin Hospital, No. 406, Jiefangnan Road, Tianjin City, 300211 China; 2 Department of Spine Surgery, Tianjin Union Medical Center, Tianjin, China; 3 Department of Emergency internal medicine, Tianjin Hospital, Tianjin, China

**Keywords:** HCP5, miR-93-5p, HMGA2, Diabetic nephropathy, High glucose

## Abstract

**Background:**

Long non-coding RNAs (lncRNAs) are widely reported to be involved in the development of human diseases. HLA complex P5 (HCP5) deregulation is associated with various diseases. However, the function of HCP5 in diabetic nephropathy (DN) is unclear.

**Methods:**

Human glomerular mesangial cells (HGMCs) were treated with high glucose (HG) to establish DN cell models. The expression of HCP5, miR-93-5p and high mobility group AT-hook 2 (HMGA2) mRNA was detected using quantitative polymerase chain reaction (QPCR). Cell proliferation and cell apoptosis were assessed using cell counting kit-8 (CCK-8) assay and flow cytometry assay, respectively. The expression of apoptosis- and fibrosis-related proteins and HMGA2 protein was quantified by western blot. The release of pro-inflammatory factor was checked using enzyme-linked immunosorbent assay (ELISA). The predicted relationship between miR-93-5p and HCP5 or HMGA2 was verified using dual-luciferase reporter assay, pull-down assay or RNA immunoprecipitation (RIP) assay.

**Results:**

The expression of HCP5 and HMGA2 was enhanced, while the expression of miR-93-5p was declined in DN serum samples and HG-treated HGMCs. HCP5 knockdown or miR-93-5p restoration ameliorated HG-induced HGMC proliferation, fibrosis and inflammation. MiR-93-5p was a target of HCP5, and miR-93-5p inhibition reversed the effects caused by HCP5 knockdown. Moreover, HMGA2 was a target of miR-93-5p, and HMGA2 overexpression abolished the effects of miR-93-5p restoration. HCP5 knockdown inhibited the AKT/mTOR signaling pathway.

**Conclusion:**

HCP5 was implicated in DN progression by modulating the miR-93-5p/HMGA2 axis, which provided new insights into the understanding of DN pathogenesis.

## Background

Diabetic nephropathy (DN), which affects approximately one-third of diabetic patients, is the main cause of end-stage renal failure, with increasing incidence [1]. Patients with DN have a high cardiovascular risk, which is equivalent to that of patients with coronary heart disease [2]. Dysfunction of mesangial cells contributes to DN development, including mesangial cell excessive proliferation and fibrosis, caused by the accumulating of extracellular matrix (ECM) components, such as collagen type I, III, IV, and fibronectin [3, 4]. In addition, inflammatory response is also an important pathophysiological feature in the development of DN [5]. The pathogenesis of DN is complex, and the mechanism of mesangial cell dysfunction is not fully understood. Therefore, it is necessary to explore new mechanisms to prevent the development of DN.

Long non-coding RNA (lncRNA), a kind of non-coding RNA molecule, plays a crucial role in the pathogenesis of human diseases, including DN. Some lncRNAs are identified to be risk factors of DN, participating in crucial processes in DN progression [6], such as mitochondrial bioenergetics, metabolic changes and endoplasmic reticulum stress [7–9]. High glucose (HG)-induced mesangial cells are widely used as DN cell models in previous studies, and numerous lncRNAs have been shown to regulate proliferation, fibrosis and inflammatory responses in HG-induced mesangial cells [10, 11], hinting that lncRNAs are essential regulators in mesangial cell dysfunctions. LncRNA HLA complex P5 (HCP5) was a well-acknowledged oncogenic driver in various cancers [12, 13]. Besides, HCP5 was proposed to be one of the potential prognostic biomarkers for predicting the risk of chronic kidney disease (CKD) [14]. However, no studies reported the role of HCP5 in DN. Our study was the first to investigate the role of HCP5 in mesangial cell dysfunctions to understand the role of HCP5 in DN progression.

The molecular functions of lncRNAs are multiple, including the regulation of microRNAs (miRNAs). MiRNAs have an extensive role in modulating the pathogenesis of several diseases by regulating their downstream molecules via binding to the 3′ untranslated region (3’UTR) of target genes [15]. MiR-93-5p is shown as a potential target of HCP5 by the bioinformatics analysis, and its role in renal interstitial fibrosis in DN has been reported [16], suggesting that miR-93-5p is involved in DN progression. However, the interplays between HCP5 and miR-93-5p were not clarified in DN, and it should be disclosed whether HCP5 mediated mesangial cell dysfunctions by regulating miR-93-5p.

High mobility group AT-hook 2 (HMGA2) is one of the small DNA-binding proteins, consisting of three “AT-hook” DNA-binding motifs [17]. It was previously reported that miRNA Let-7d blocked TGF-β1-induced renal fibrogenesis in DN by decreasing the expression of HMGA2 [18], suggesting that HMGA2 might be a risk factor for DN. Bioinformatics analysis presents that miR-93-5p binds to HMGA2 3’UTR. Nevertheless, the relationship between miR-93-5p and HMGA2 is not confirmed.

Herein, we construct DN cell models by treating human glomerular mesangial cells (HGMCs) with HG and examined the expression of HCP5, miR-93-5p and HMGA2 in DN serum samples and cell models. Functionally, we for the first time investigated the role of HCP5 on proliferation, fibrosis and inflammatory responses in HG-treated HGMCs. Moreover, we provided a new mechanism for HCP5 to participate in DN progression.

## Methods

### Serum samples

A total of 28 DN patients and 28 normal subjects were recruited from Tianjin Hospital.

Patients with DN were enrolled according to the following inclusion criteria: diagnosis of diabetes mellitus with concomitant macroalbuminuria, or the duration of diabetes mellitus more than 10 years with concomitant microalbuminuria. The clinical characteristics of DN patients and normal subjects, such as gender, age, body mass index (BMI) and etc., were displayed in Table 1. Blood samples were collected from these DN patients and normal subjects with written informed consent by all participants. Blood samples were subjected to centrifugation to obtain serum samples that were preserved at − 80 °C conditions until use. This study was carried out with the permission of the Ethics Committee of Tianjin Hospital.

**Table 1:** Clinical characteristics of patients with diabetic nephropathy and healthy controls

Characteristic	Normal	DN
Male/female (number)		
Male	15	18
Female	13	10
Age (y)	51.3 ± 5.2	50.7 ± 7.8
BMI (kg/m2)	22.8 ± 2.8	24.2 ± 3.7
SBP (mm Hg)	109.9 ± 15.3	156.2 ± 27.7*
DBP (mm Hg)	80.1 ± 6.8	85.8 ± 10.2*
FPG (mM)	4.9 ± 2.2	8.3 ± 2.3*
HbA1c (%)	5.2 ± 1.8	8.2 ± 2.1*
TC (mM)	4.98 ± 1.2	6.13 ± 1.5*
TG (mM)	1.26 ± 1.1	2.36 ± 1.21*
LDL-C (mM)	3.05 ± 1.12	3.68 ± 1.16*
BUN (mM)	5.19 ± 1.32	8.26 ± 1.41*
SCr (μM)	60.18 ± 11.35	121.08 ± 20.23*

Abbreviations: BMI, body mass index; BUN, blood urea nitrogen; DBP, diastolic blood pressure; DN, diabetic nephropathy; FPG, fasting plasma glucose; LDL-C, low-density lipoprotein cholesterol; SBP, systolic blood pressure; SCr, serum creatinine; TC, total cholesterol; TG, triglycerides. Unless indicated otherwise, data are given as the mean ± SD. *P < 0.05

### Cells and DN cell model

HGMCs were purchased from Procell (CP-H067; Wuhan, China) and maintained in matched dedicated complete culture medium (CM-H067; Procell) at 37 °C atmosphere of 5% CO_2_. To induce DN cell model, HGMCs were cultured in HG culture medium (supplementing with D-glucose at a final concentration of 25 mmol/L) [19]. HGMCs cultured in normal glucose (NG) culture medium (supplementing with 5.5 mmol/L D-glucose) were used as the control.

### Quantitative polymerase chain reaction (QPCR)

Total RNA was extracted from serum or cells using TRIzol reagent (Invitrogen, Carlsbad, CA, USA). For HCP5 and HMGA2, cDNA reverse transcription was performed using the High Capacity cDNA Reverse Transcription kit (Applied Biosystem, Foster city, CA, USA), and QPCR was performed using SYBR Green PCR Master Mix (Applied Biosystem). For miR-93-5p, miRNAs were reverse transcribed into cDNA using a Mir-X miRNA first-strand synthesis kit (Takara, Dalian, China), followed by QPCR using SYBR Green PCR Master Mix (Applied Biosystem). The housekeeping gene GAPDH or U6 was used as the endogenous control. Relative expression was presented using the 2^−ΔΔCt^ method. The primers used were listed in Table 2.

**Table 2:** The sequences of primers for qRT-PCR

Name	Accession number	Product size	Sequences (5′-3′)
HCP5	NR_040662	241 bp	F: 5′-GACTCTCCTACTGGTGCTTGGT-3′
			R: 5′-CACTGCCTGGTGAGCCTGTT-3′
miR-93-5p	MIMAT0000093	67 bp	F: 5′-CGCAAAGTGCTGTTCGTGC-3’
			R: 5′-AGTGCAGGGTCCGAGGTATT-3’
GAPDH	NM_002046	209 bp	F: 5′-GATGCTGGCGCTGAGTACG-3’
			R: 5′-GCTAAGCAGTTGGTGGTGC-3’
HMGA2	NM_003483	137 bp	F: 5′-TTCAGCCCAGGGACAACCT-3’
			R: 5′-TCTTGTTTTTGCTGCCTTTGG-3’
U6	NR_004394	94 bp	F: 5′-CTCGCTTCGGCAGCACATA-3’
			R: 5′-AACGATTCACGAATTTGCGT-3’

### Oligonucleotides and plasmids

Small interference RNA (siRNA) against HCP5 (si-HCP5; Ribobio, Guangzhou, China) was used for HCP5 knockdown, with siRNA negative control (si-NC) as control. MiR-93-5p mimic (miR-93-5p; Ribobio) was used for miR-93-5p enrichment, and miR-93-5p inhibitor (anti-miR-93-5p; Ribobio) was used for miR-93-5p inhibition, with mimic negative control (miR-NC) or inhibitor negative control (anti-NC) as control. HMGA2 overexpression plasmid, pcDNA-HMGA2 (HMGA2), was purchased from Sangon Biotech (Shanghai, China), with empty vector as control. These plasmids or oligonucleotides were transfected into HGMCs using the Lipofectamine 3000 transfection kit (Invitrogen).

### Cell counting kit-8 (CCK-8) test

CCK-8 assay for cell proliferation detection was performed using CCK-8 reagent (Dojindo, Kumamoto, Japan). Cells were seeded into 96-well plates at a density of 2000 cells/well. After culturing for 24, 48 and 72 h, cells were treated with 10 μL CCK-8 reagent for 2 h. Cell viability was detected according to the absorbance at 450 nm using a microplate reader (Thermo Fisher Scientific, Waltham, MA, USA).

### Flow cytometry assay

Treated or transfected cells were cultured for 48 h with serum-deprivation to induce cell apoptosis. Then cells were harvested and washed with phosphate buffered saline (PBS). Cells suspended in Annexin V-FIFC binding buffer (Beyotime, Shanghai, China) were incubated with Annexin V-FIFC (Beyotime) and propidium iodide (PI; Beyotime) in line with the protocol. Cell apoptosis was next distinguished using a flow cytometer (Beckman, Miami, CA, USA).

### Western blot assay

Total protein from cells was extracted using RIPA lysis buffer (Thermo Fisher Scientific). The protein was denatured and then separated by 10% SDS-PAGE electrophoresis. The separated proteins were transferred into a PVDF membrane and subsequently blocked using 5% skim milk. The protein-stained membranes were probed with specific primary and secondary antibodies. Antibodies against Cleaved-PARP (ab32064), Cleaved-caspase 3 (ab32042), FN (ab2413), Col IV (ab86042), Col I (ab138492), HMGA2 (ab97276) and GAPDH (AB9485) were purchased from Abcam (Cambridge, MA, USA). The signals of protein blots were captured using the enhanced chemiluminescence kit (Beyotime), and the densitometry of the blots was quantified using ImageJ software (NIH, Bethesda, MA, USA).

### ELISA

The release of pro-inflammatory factors, including TNF-α, IL-6 and IL-1β, was checked using the matched ELISA kits, including Human TNF alpha ELISA Kit (Abcam), Human IL-6 ELISA Kit (Abcam) and Human IL-1β ELISA Kit (Abcam). The experimental procedures were conducted in line with the guidelines.

### Dual-luciferase reporter assay

The relationship between miR-93-5p and HCP5 or HMGA2 was predicted by starbase (http://starbase.sysu.edu.cn/), which provided the binding site between them. The wild-type (WT) and mutant-type (MUT) sequence fragment of HCP5 and HMGA2 3’UTR were cloned into PGL4 reporter plasmid, respectively. Fusion plasmids were named as HCP5-WT, HCP5-MUT, HMGA2–3’UTR-WT and HMGA2–3’UTR-MUT. HGMCs were transfected with miR-93-5p mimic and HCP5-WT, HCP5-MUT, HMGA2–3’UTR-WT and HMGA2–3’UTR-MUT, with miR-NC as control. After incubating for 48 h, cells were collected to detect the luciferase activity using the Dual-Luciferase Assay System (Promega, Madison, WI, USA).

### Pull-down assay

Biotin-labeled miR-93-5p probe (Bio-miR-93-5p) and oligo negative control probe (Bio-NC) were purchased from Ribobio. HGMCs were transfected with Bio-miR-93-5p or Bio-NC and treated with lysis buffer from the Pierce™ Magnetic RNA-Protein Pull-Down Kit (Thermo Fisher Scientific). Cell lysates were incubated with streptavidin magnetic beads. Then the biotin-coupled RNA complex was pulled down, and the abundance of HCP5 or HMGA2 in the pull-down compounds was checked by QPCR.

### RNA immunoprecipitation (RIP) assay

By using the EZMagna RIP kit (Millipore, Billerica, MA, USA), HGMCs were exposed to lysis buffer, and the lysates were incubated with the magnetic beads pre-conjugated with Argonaute2 antibody (anti-Ago2; Millipore) or Immunoglobulin G antibody (anti-IgG; Millipore). RNAs bound to beads were eluted, purified and used for QPCR to detect the expression of HCP5.

### Statistical analysis

All experiments were performed in triplicate. The data from triplicate biological replicates were shown as mean ± standard deviation (SD). All statistical analyses were executed using GraphPad Prism 7.0 (GraphPad, Inc., La Jolla, CA, USA). The Kolmogorov-Smirnov test was used to test the data for normality. For normally distributed data, the difference between two groups was analyzed by Student’s *t*-test, and the difference among multiple groups was analyzed by analysis of variance (ANOVA) and Tukey post hoc test. Pearson correlation coefficient was used to assess the correlation between two sets. *P*-value of less than 0.05 was considered to be statistically significant.

## Results

### The expression of HCP5 was elevated, while the expression of miR-93-5p was declined in serum samples from DN patients and HG-administered HGMCs

**Fig. 1:**
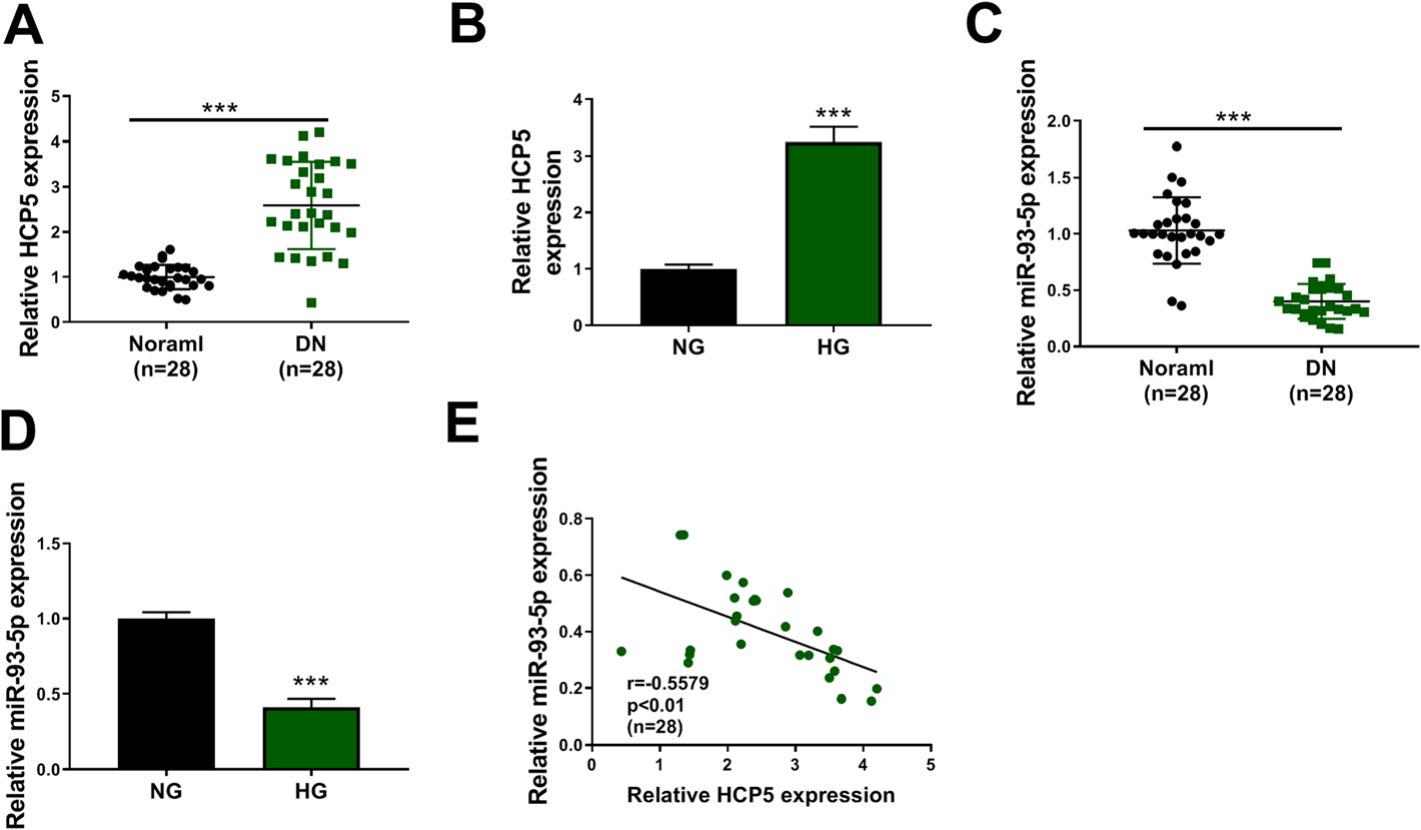
HCP5 was upregulated, while miR-93-5p was downregulated in DN serum samples and HG-treated HGMCs. **A** The expression of HCP5 in serum samples from DN patients and normal subjects was detected by QPCR. **B** The expression of HCP5 in HG- and NG-treated HGMCs was detected by QPCR. **C** The expression of miR-93-5p in serum samples from DN patients and normal subjects was measured by QPCR. **D** The expression of miR-93-5p in HG- and NG-treated HGMCs was measured by QPCR. **E** The correlation between miR-93-5p expression and HCP5 expression in DN serum samples was determined by Pearson correlation coefficient. ***P < 0.001

We examined the expression of HCP5 and miR-93-5p in DN serum samples and DN cell models. As shown in Fig. 1A, HCP5 was notably upregulated in serum samples from DN patients compared to normal subjects. Also, HCP5 was highly expressed in HG-administered HGMCs compared with that in NG-treated HGMCs (Fig. 1B). Inversely, the expression of miR-93-5p was notably decreased in serum samples from DN patients and HG-administered HGMCs compared to normal subjects and NG-induced HGMCs, respectively (Fig. 1C and D). Besides, miR-93-5p expression was negatively correlated with HCP5 expression in DN serum samples (Fig. 1E). The data showed that the expression patterns of HCP5 and miR-93-5p were opposite in DN serum samples and cell models.

### HCP5 knockdown alleviated HG-induced proliferation, fibrosis and inflammation of HGMCs

**Fig. 2:**
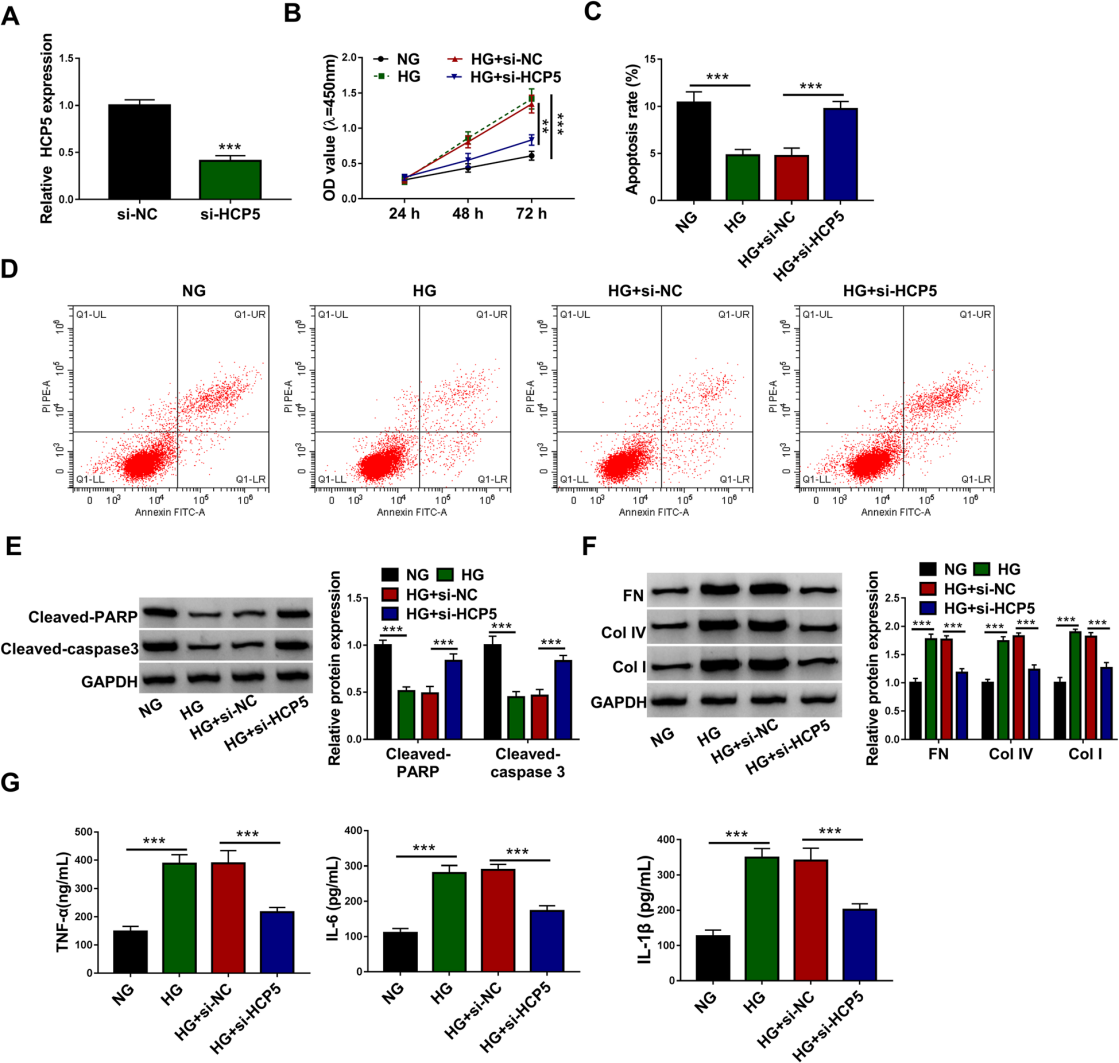
HCP5 knockdown alleviated HG-induced HGMC dysfunctions. **A** The transfection of si-HCP5 notably diminished the expression of HCP5 by QPCR analysis. In HG, NG, HG + si-HCP5 and HG + si-NC groups, (**B**) cell proliferation was assessed by CCK-8 assay, and (**C** and **D**) cell apoptosis was evaluated by flow cytometry assay. **E** Cell apoptosis was also assessed by the protein levels of Cleaved-PARP and Cleaved-caspase 3 using western blot. **F** Cell fibrosis was assessed by the levels of FN, Col IV and Col I using western blot. **G** The release of TNF-α, IL-6 and IL-1β was examined using ELISA kits. ***P < 0.001

The above data showed a high expression level of HCP5 in HG-administered HGMCs. We thus reduced the expression of HCP5 to explore its potential effects. After si-HCP5 transfection, the expression of HCP5 was strikingly decreased (Fig. 2A), suggesting that si-HCP5 was available. We examined cell proliferation using CCK-8 assay, and the data showed that HG significantly stimulated the proliferation of HGMCs compared to NG, while HCP5 knockdown relieved HG-induced proliferation (Fig. 2A). To determine whether HG-induced proliferation of HGMCs was mediated partly by apoptosis, we performed flow cytometry assay to assess cell apoptosis. In our data, the apoptosis rate was notably inhibited by HG but enhanced by HCP5 knockdown (Fig. 2C and D). Cleaved-PARP and Cleaved-caspase 3 were well-known markers during apoptosis signaling. The levels of Cleaved-PARP and Cleaved-caspase 3 were decreased in HG-treated HGMCs, while the levels of Cleaved-PARP and Cleaved-caspase 3 were recovered by HCP5 knockdown (Fig. 2E). Besides, cell fibrosis-related markers, including FN, Col IV and Col I, were all highly expressed in HG-treated HGMCs, while their levels were repressed by HCP5 knockdown (Fig. 2F). Moreover, we found HG promoted the release of TNF-α, IL-6 and IL-1β, leading to inflammatory responses, while HCP5 knockdown inhibited the release of these inflammatory factors (Fig. 2G). Overall, HCP5 knockdown alleviated HG-induced excessive proliferation, fibrosis and inflammation of HGMCs.

### Cytoplasmic HCP5 functioned as miR-93-5p sponge

**Fig. 3:**
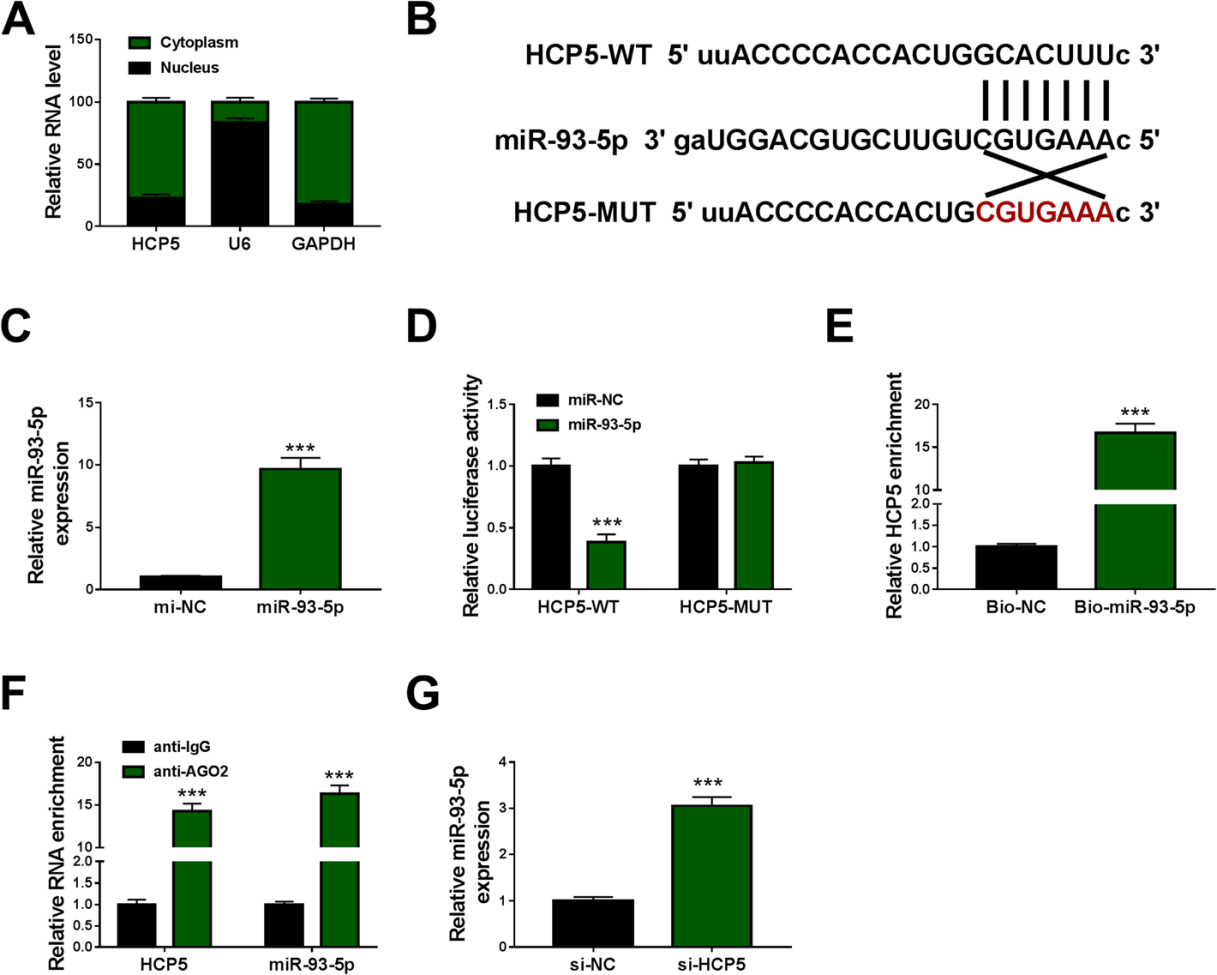
HCP5 bound to miR-93-5p. **A** The distribution of HCP5 in the cytoplasm or nucleus was examined using QPCR. **B** The binding site between HCP5 and miR-93-5p. **C** The availability of miR-93-5p mimic was checked using QPCR. The relationship between HCP5 and miR-93-5p was verified by (**D**) dual-luciferase reporter assay, (**E**) pull-down assay and (**F**) RIP assay. **G** The expression of miR-93-5p in HGMCs transfected with si-HCP5 or si-NC was detected by QPCR. ***P < 0.001

Subcellular distribution analysis showed that HCP5 was mainly located in the cytoplasm but not in the nucleus (Fig. 3A). MiR-93-5p was predicted as a target of HCP5 by starbase. Then, we performed experiments to verify the relationship between HCP5 and miR-93-5p. The wild-type and mutant-type of HCP5 dual-luciferase reporter plasmids were constructed (Fig. 3B). The expression of miR-93-5p was strikingly enhanced in HGMCs transfected with miR-93-5p compared to miR-NC (Fig. 3C). In miR-93-5p-overexpressed HGMCs, the transfection of HCP5-WT significantly weakened the luciferase activity, while the transfection of HCP5-MUT hardly changed the luciferase activity (Fig. 3D). Besides, Bio-miR-93-5p could pull a high abundance of HCP5 down in pull-down assay (Fig. 3E). In addition, both HCP5 and miR-93-5p could be abundantly detected in the anti-AGO2-conjugated beads compared with that in the anti-IgG-conjugated beads in RIP assay (Fig. 3F). Moreover, the expression of miR-93-5p was largely promoted in HGMCs transfected with si-HCP5 (Fig. 3G). Collectively, all findings claimed that miR-93-5p was a target of HCP5.

### MiR-93-5p ameliorated HG-induced proliferation, fibrosis and inflammation of HGMCs

**Fig. 4:**
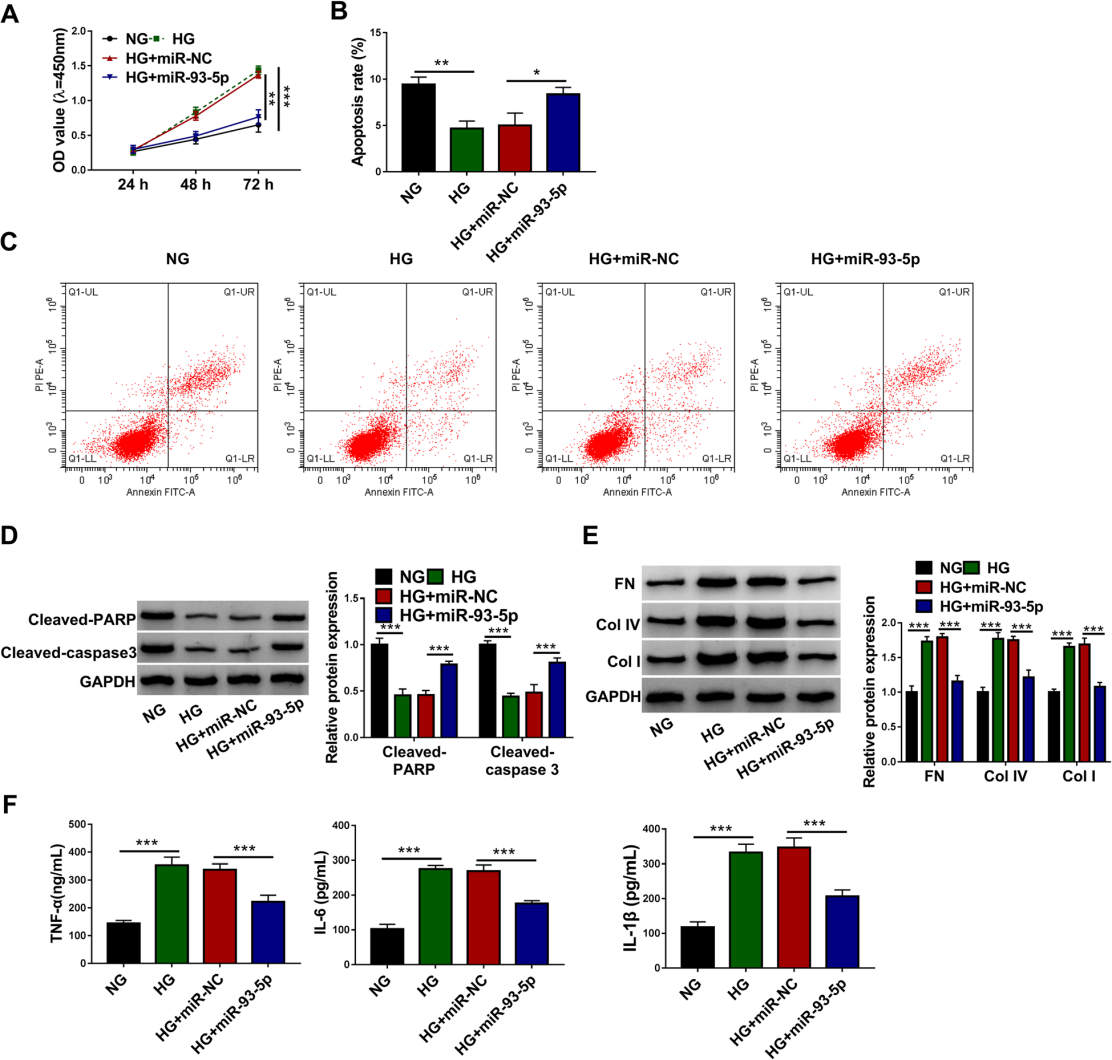
MiR-93-5p restoration alleviated HG-induced HGMC dysfunctions. In the NG, HG, HG + miR-93-5p and HG + miR-NC groups, (**A**) cell proliferation was assessed by CCK-8 assay. **B** and **C** Cell apoptosis was investigated using flow cytometry assay. **D** The protein levels of Cleaved-PARP and Cleaved-caspase 3 were detected using western blot. **E** The protein levels of FN, Col IV and Col I were detected by western blot. **F** The release of TNF-α, IL-6 and IL-1β was examined using ELISA kits. *P < 0.05, **P < 0.01 and ***P < 0.001

We next investigated the role of miR-93-5p in DN cell models. HG-induced HGMC excessive proliferation was repressed by miR-93-5p restoration (Fig. 4A). Besides, HG-restrained cell apoptosis was partly promoted by miR-93-5p overexpression (Fig. 4B and C), which was further verified by the protein levels of Cleaved-PARP and Cleaved-caspase 3. The data presented that the levels of Cleaved-PARP and Cleaved-caspase 3 repressed in HG-induced HGMCs were stimulated by the reintroduction of miR-93-5p (Fig. 4D). In addition, the protein levels of FN, Col IV and Col I were weakened by miR-93-5p transfection in HG-treated HGMCs (Fig. 4E). Additionally, the release of TNF-α, IL-6 and IL-1β stimulated by HG was largely blocked by miR-93-5p restoration (Fig. 4F). The data indicated that miR-93-5p suppressed HG-induced excessive proliferation, fibrosis and inflammation of HGMCs.

### MiR-93-5p inhibition recovered HCP5 knockdown-suppressed excessive proliferation, fibrosis and inflammation in HG-treated HGMCs

**Fig. 5:**
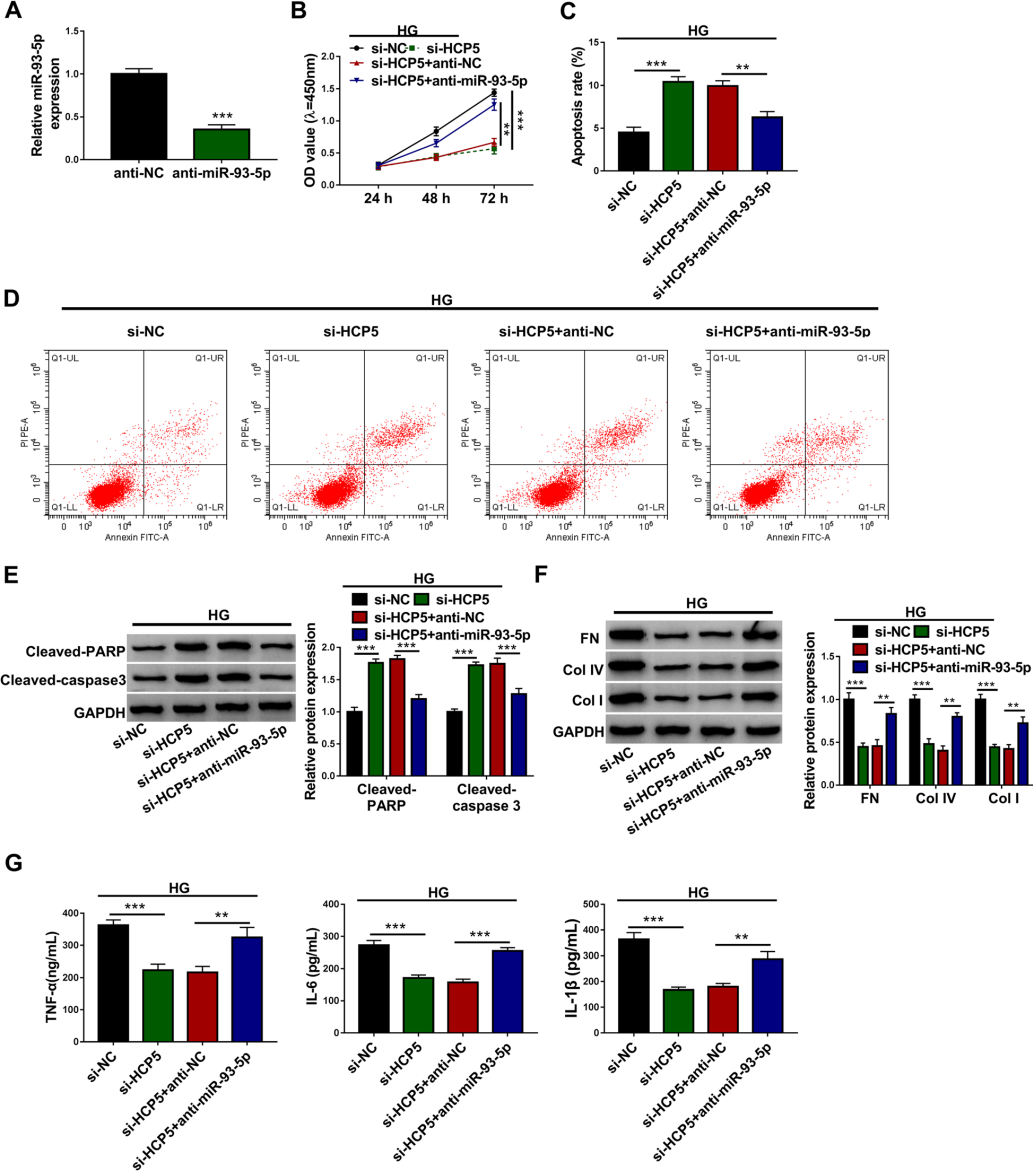
MiR-93-5p inhibition reversed the effects of HCP5 knockdown. **A** The availability of miR-93-5p inhibitor was checked using QPCR. In the si-NC, si-HCP5, si-HCP5 + anti-NC and si-HCP5 + anti-NC groups, (**B**) cell proliferation was checked using CCK-8 assay. **C** and **D** Cell apoptosis was analyzed by flow cytometry assay. **E** The levels of Cleaved-PARP and Cleaved-caspase 3 were quantified using western blot. **F** The protein levels of FN, Col IV and Col I were detected by western blot. **G** The release of TNF-α, IL-6 and IL-1β was determined using ELISA kits. **P < 0.01 and ***P < 0.001

We subsequently performed rescue experiments to determine the interaction between HCP5 and miR-93-5p in function. The expression of miR-93-5p was remarkably decreased in HGMCs transfected with anti-miR-93-5p (Fig. 5A). Then, HG-treated HGMCs were transfected with si-HCP5, si-NC, si-HCP5 + anti-miR-93-5p or si-HCP5 + anti-NC. We found only si-HCP5 transfection blocked cell proliferation, while si-HCP5 + anti-miR-93-5p cotransfection largely restored cell proliferation (Fig. 5B). HCP5 knockdown-stimulated apoptosis in HG-treated HGMCs was partly repressed by miR-93-5p inhibition (Fig. 5C and D). The levels of Cleaved-PARP and Cleaved-caspase 3 were promoted in HG-treated HGMCs transfected with si-HCP5 alone but lessened in HG-treated HGMCs transfected with si-HCP5 + anti-miR-93-5p (Fig. 5E). Moreover, the levels of FN, Col IV and Col I suppressed in si-HCP5-transfected HG-treated HGMCs were largely recovered by the reintroduction of anti-miR-93-5p (Fig. 5F). The release of TNF-α, IL-6 and IL-1β was also blocked by si-HCP5 but stimulated by si-HCP5 + anti-miR-93-5p in HG-treated HGMCs (Fig. 5G). Overall, HCP5 knockdown increased the expression of miR-93-5p to inhibit HG-induced dysfunctions in HGMCs.

### HMGA2 was a target of miR-93-5p

**Fig. 6:**
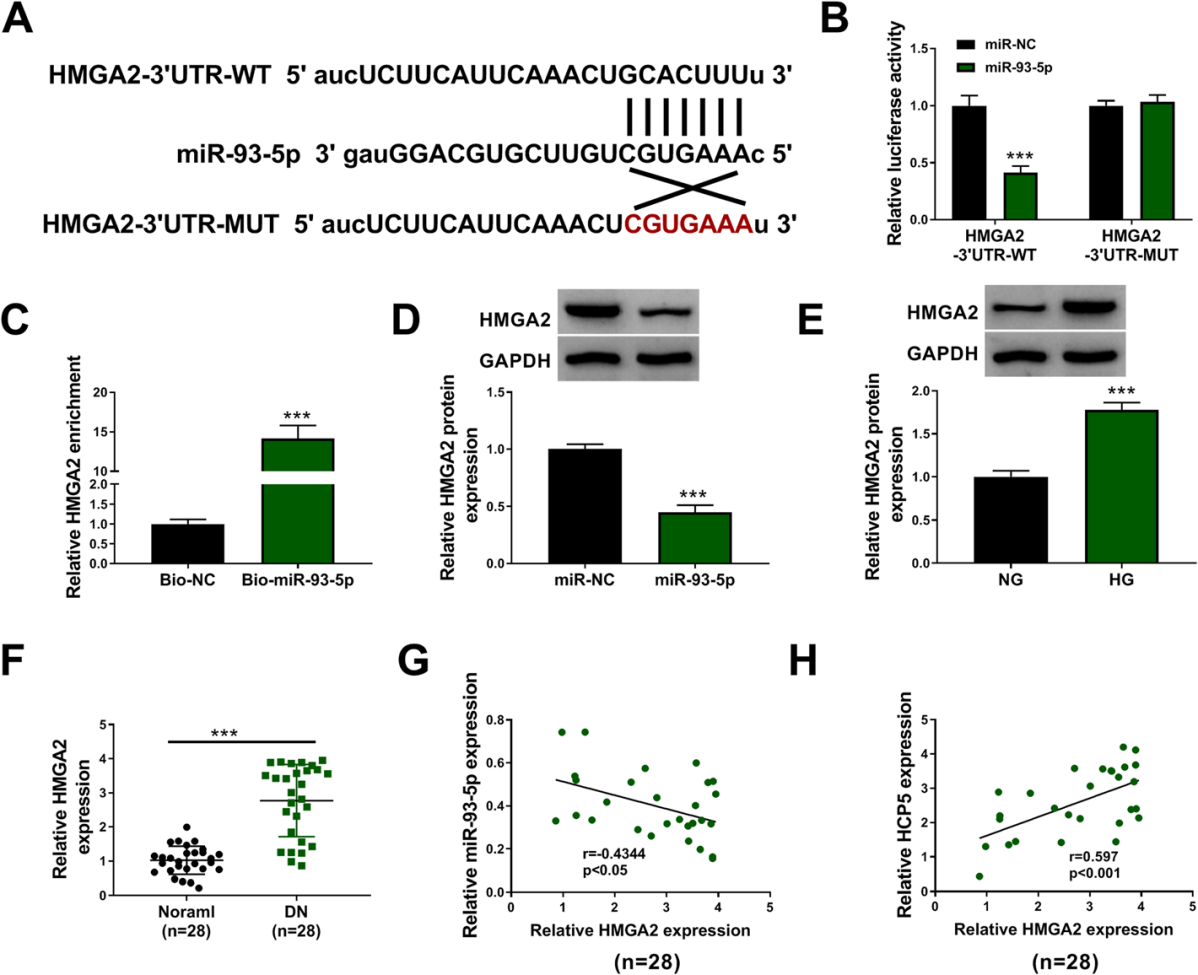
MiR-93-5p bound to HMGA2 3’UTR. **A** The binding site between HMGA2 and miR-93-5p. The relationship between miR-93-5p and HMGA2 was confirmed using (**B**) dual-luciferase reporter assay and (**C**) pull-down assay. **D** The expression of HMGA2 protein in HGMCs transfected with miR-93-5p or miR-NC was detected by western blot. **E** The expression of HMGA2 protein in HG- and NG-treated HGMCs was detected by western blot. **F** The expression of HMGA2 in serum samples from DN patients and normal subjects was measured by QPCR. **G** and **H** The correlation between HMGA2 expression and miR-93-5p expression or HCP5 expression in DN serum samples was determined by Pearson correlation coefficient. ***P < 0.001

Bioinformatics tool starbase showed that HMGA2 was a potential target of miR-93-5p, and miR-93-5p interacted with HMGA2 3’UTR. We then constructed the wild-type and mutant-type reporter plasmids of HMGA2 to conduct dual-luciferase reporter assay (Fig. 6A). The results displayed that HGMCs transfected with miR-93-5p and HMGA2–3’UTR-WT showed a decrease of luciferase activity (Fig. 6B). Besides, Bio-miR-93-5p could enrich a high abundance of HMGA2 compared to Bio-NC (Fig. 6C). Moreover, the expression of HMGA2 protein was markedly suppressed by miR-93-5p overexpression in HGMCs (Fig. 6D). In short, sufficient evidence indicated that HMGA2 was a target of miR-93-5p. The expression of HMGA2 protein was strikingly enhanced in HG-treated HGMCs compared to NG (Fig. 6E). The expression of HMGA2 mRNA was also notably elevated in serum samples from DN patients compared with that from normal subjects (Fig. 6F). In these DN serum samples, we ensured that HMGA2 expression was negatively correlated with miR-93-5p expression but positively correlated with HCP5 expression (Fig. 6G and H).

### MiR-93-5p restoration weakened the expression of HMGA2 to suppress excessive proliferation, fibrosis and inflammation in HG-treated HGMCs

**Fig. 7:**
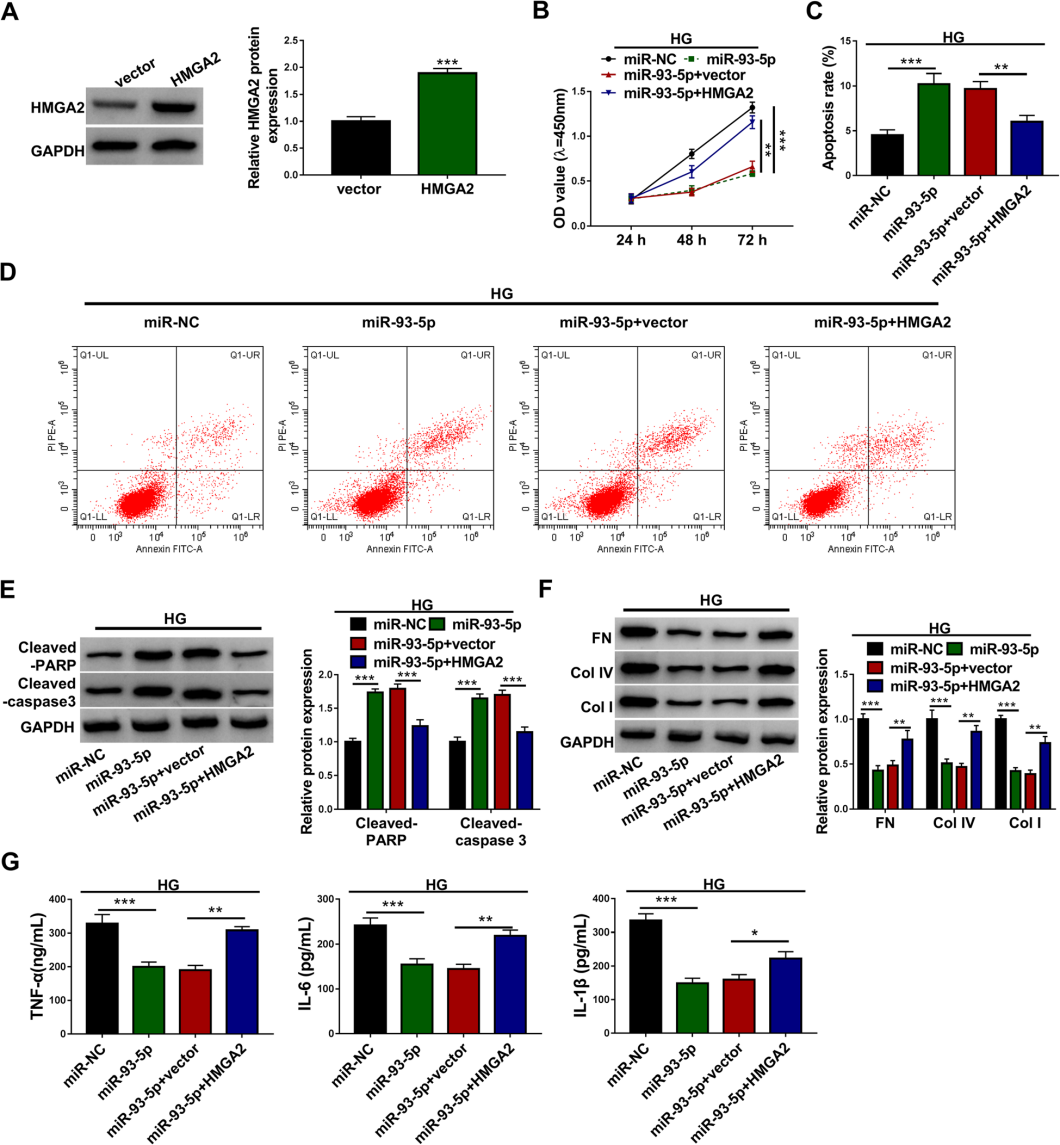
HMGA2 overexpression abolished the effects of mIR-93-5p restoration. **A** The availability of HMGA2 overexpression vector was checked using western blot. In the miR-NC, miR-93-5p, miR-93-5p + vector and miR-93-5p + HMGA2 groups, (**B**) cell proliferation was monitored using CCK-8 assay. **C** and **D** Cell apoptosis was investigated using flow cytometry assay. **E** The levels of Cleaved-PARP and Cleaved-caspase 3 were quantified using western blot. **F** The protein levels of FN, Col IV and Col I were checked by western blot. **G** The release of TNF-α, IL-6 and IL-1β was ascertained using ELISA kits. *P < 0.05, **P < 0.01 and ***P < 0.001

We next performed rescue experiments to determine the interaction between miR-93-5p and HMGA2 in function. The expression of HMGA2 protein was strikingly promoted in HGMCs transfected with HMGA2 compared to vector (Fig. 7A). Then, HG-treated HGMCs were transfected with miR-93-5p, miR-NC, miR-93-5p + HMGA2 and miR-93-5p + vector. HG-stimulated cell proliferation was significantly blocked by miR-93-5p restoration but recovered by the reintroduction of HMGA2 (Fig. 7B). HG-blocked cell apoptosis was notably promoted by miR-93-5p but repressed by the reintroduction of HMGA2 (Fig. 7C and D). The levels of Cleaved-PARP and Cleaved-caspase 3 proteins were reinforced in HG-treated HGMCs transfected with miR-93-5p but repressed in HG-treated HGMCs transfected with miR-93-5p + HMGA2 (Fig. 7E). The levels of FN, Col IV and Col I proteins were notably suppressed in HG-treated HGMCs transfected with miR-93-5p but largely recovered in HG-treated HGMCs transfected with miR-93-5p + HMGA2 (Fig. 7F). Furthermore, miR-93-5p restoration blocked the release of TNF-α, IL-6 and IL-1β, while HMGA2 reintroduction restored the release of these pro-inflammatory factors (Fig. 7G). Overall, miR-93-5p restoration suppressed excessive proliferation, fibrosis and inflammation of HG-treated HGMCs by degrading HMGA2.

### HCP5-mediated miR-93-5p/HCP5 axis regulated the activity of the AKT/mTOR signaling pathway

**Fig. 8:**
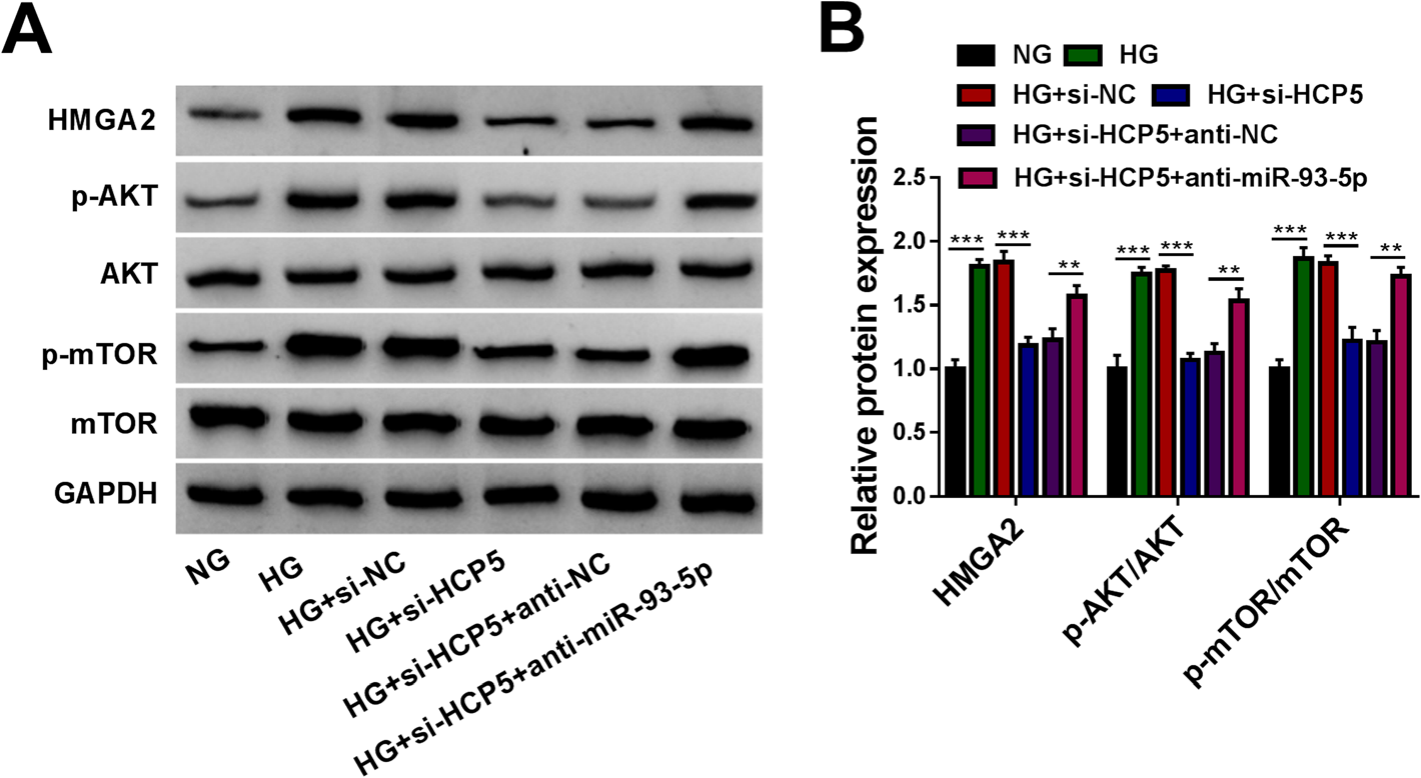
HCP5/miR-93-5p/HCP5 axis regulated HG-induced HGMC dysfunctions maybe by repressing the AKT/mTOR signaling pathway. (**A** and **B**) In NG, HG, HG + si-NC, HG + si-HCP5, HG + si-HCP5 + anti-NC and HG + si-HCP5 + anti-miR-93-5p groups, the expression of HMGA2, p-AKT and p-mTOR proteins was quantified by western blot. **P < 0.01 and ***P < 0.001

Further expression analyses uncovered that the expression of HMGA2 protein in HG-treated HGMCs was significantly inhibited by si-HCP5 transfection but largely recovered by si-HCP5 + anti-miR-93-5p transfection (Fig. 8A and B). Interestingly, we found that the expression levels of p-AKT and p-mTOR proteins were pronouncedly strengthened by HG, while si-HCP5 transfection notably weakened the expression levels of p-AKT and p-mTOR (Fig. 8A and B). In addition, the si-HCP5 + anti-miR-93-5p cotransfection markedly recovered the expression of p-AKT and p-mTOR proteins in HG-treated HGMCs (Fig. 8A and B), suggesting that HCP5 knockdown inhibited the AKT/mTOR signaling pathway by governing the miR-93-5p/HCP5 axis.

## Discussion

Our present study mainly discovered that the expression of HCP5 was aberrantly upregulated in DN serum samples and HG-treated HGMCs. HCP5 knockdown inhibited HG-induced dysfunctions of HGMCs, including excessive proliferation, ECM accumulation and inflammatory responses. In mechanism, we identified that HCP5 competitively bound to miR-93-5p, in turn increased the expression level of HMGA2. Rescue experiments suggested that HCP5 knockdown blocked HG-induced HGMC dysfunctions by modulating the miR-93-5p/HMGA2 axis. Moreover, we found that HCP5 knockdown resulted in decreased levels of p-AKT and p-mTOR proteins, hinting that the AKT/mTOR signaling pathway was involved in HCP5-mediated regulatory network.

Numerous studies introduced that HCP5 overexpression promoted cancer cell malignant behaviors, such as epithelial-mesenchymal transition (EMT), proliferation, migration, invasion and angiogenic ability [12, 13, 20], suggesting the vital role of HCP5 in cancer initiation and progression. Besides, HCP5 expression was previously shown to be increased in CKD patients compared to normal control [14], hinting that HCP5 might participate in kidney-associated diseases. Consistently, our data, for the first time, found that the expression of HCP5 was notably enhanced in serum samples from DN patients and HG-treated HGMCs. Gain- or loss-function assays indicated that HCP5 knockdown alleviated HG-induced HGMC dysfunctions, including excessive proliferation, fibrosis and inflammation. The evidence hinted that HCP5 was a potential pathogenic factor of DN, and the targeted inhibition of HCP5 might be a new strategy in clinical application of DN treatment. However, more functions of HCP5 should be explored in future work, such as oxidative stress and endoplasmic reticulum stress.

Bioinformatics analysis predicted that HCP5 harbored miR-93-5p binding site, and their putative target relationship was validated by dual-luciferase reporter assay, pull-down assay and RIP assay. Previous study showed that miR-93-5p was markedly downregulated in patients with CDK compared to healthy controls [21]. Besides, miR-93-5p inhibition abolished the effects of lncRNA XIST knockdown, thus promoting renal interstitial fibrosis in DN mice and HG-induced human kidney cells (HK-2) [16]. Consistent with these findings, our data presented that miR-93-5p restoration inhibited HG-induced HGMC proliferation, fibrosis and inflammation, while miR-93-5p inhibition promoted these dysfunctions that were blocked by HCP5 knockdown. The results hinted that the strategy of miR-93-5p enrichment might be conductive for DN management.

Furthermore, HMGA2 was a target of miR-93-5p, and miR-93-5p bound to HMGA2 3’UTR to inhibit its expression. Our data manifested that HMGA2 expression was strengthened in DN serum samples and HG-treated HGMCs. In function, HMGA2 overexpression recovered miR-93-5p restoration-inhibited HGMC proliferation, fibrosis and inflammation, which was consistent with the findings from previous studies [18, 22, 23]. All findings highlighted that HMGA2 was a pathogenic factor of DN. Additionally, we found the expression of p-AKT and p-mTOR proteins was heightened in HG-treated HGMCs but repressed by HCP5 knockdown. We speculated that HCP5 knockdown alleviated HG-induced HGMC dysfunctions through the inactivation of the AKT/mTOR signaling pathway. Previous studies claimed that Forkhead box P1 (FOXP1) suppressed HG-induced ECM accumulation and oxidative stress in mesangial cells via inhibiting the AKT/mTOR signaling pathway [24]. LncRNA NEAT1 promoted HG-induced proliferation and fibrosis in mouse mesangial cells by activating the AKT/mTOR signaling pathway [25]. The data suggested that the activation of the AKT/mTOR signaling pathway was a vital process in DN development. Nonetheless, the involvement of the AKT/mTOR signaling pathway in the HCP5-mediated regulatory network needed further validation.

Though our study provides novel insights into understanding the pathogenesis of DN from the perspective of HCP5, there are still limitations of the present study. For example, this study is only a preliminary study of the role of HCP5 in DN in vitro, and animal models should be constructed to further these findings in vivo. These issues should be elucidated in future work.

## Conclusions

**Fig. 9:**
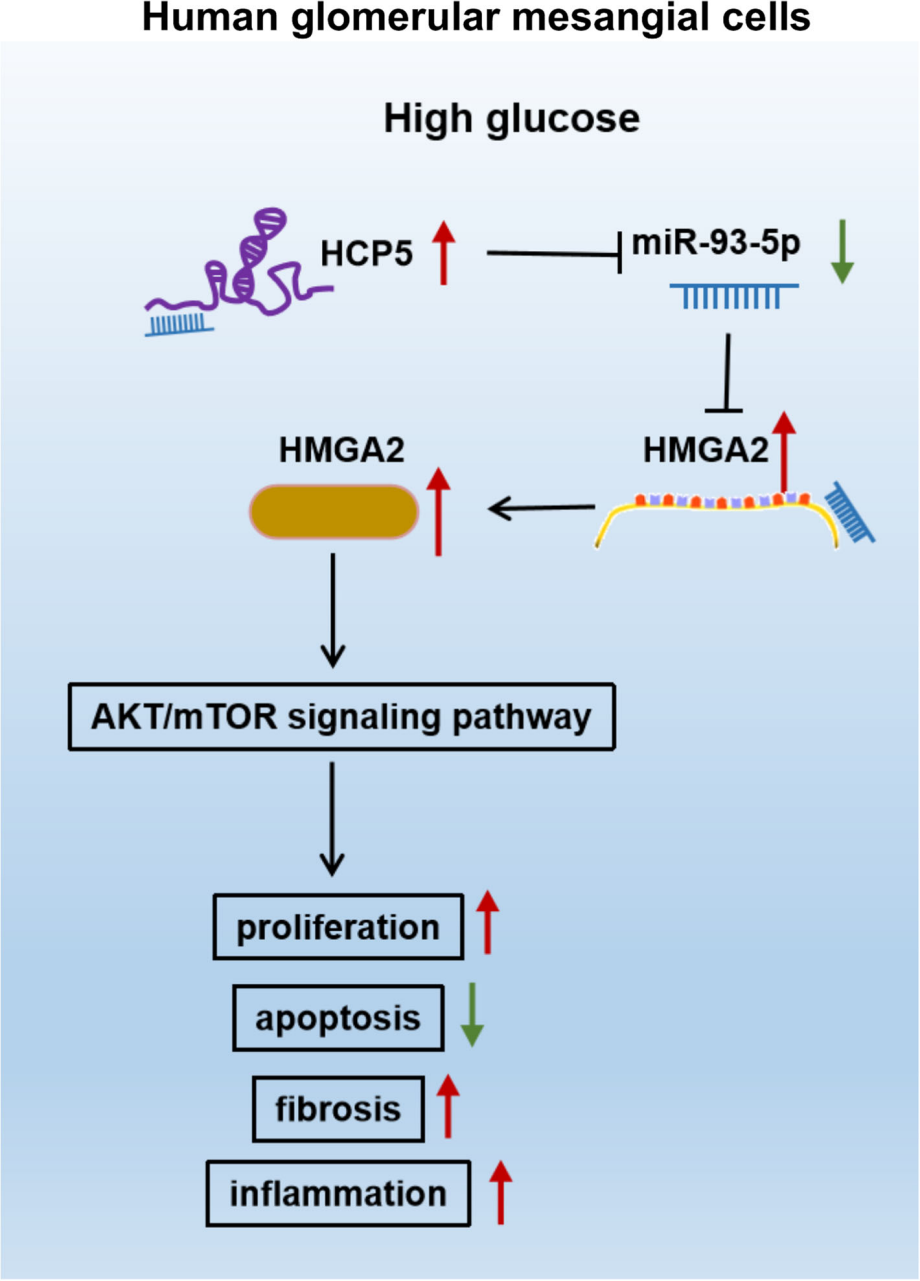
In HG-induced HGMCs, increased HCP5 functioned as miR-93-5p sponge to promote HMGA2 expression and then governed the AKT/mTOR signaling, thus regulating several cellular processes

We first explored the role of HCP5 in DN cell models and defined HCP5 as a potential pathogenic factor. In HG-induced HGMCs, we found increased HCP5 governed the miR-93-5p/HMGA2 axis to activate the AKT/mTOR pathway, thus contributing to HGMC excessive proliferation, fibrosis and inflammatory responses (Fig. 9).

## Data Availability

All data generated or analyzed during this study are included in this article.

## Abbreviations

lncRNA: long non-coding RNA
miR: microRNA
HCP5: HLA complex P5
DN: diabetic nephropathy
HGMCs: Human glomerular mesangial cells
HG: high glucose
HMGA2: high mobility group AT-hook 2
ELISA: enzyme-linked immunosorbent assay
mTOR: mammalian target of rapamycin
QPCR: quantitative polymerase chain reaction
CCK-8: cell counting kit-8
RIP: RNA immunoprecipitation

## References

[1] Dounousi E, Duni A, Leivaditis K, Vaios V, Eleftheriadis T, Liakopoulos V (2015). Improvements in the Management of Diabetic Nephropathy. Rev Diabet Stud.

[2] Tonelli M, Muntner P, Lloyd A, Manns BJ, Klarenbach S, Pannu N, James MT, Hemmelgarn BR (2012). Risk of coronary events in people with chronic kidney disease compared with those with diabetes: a population-level cohort study. Lancet..

[3] Shemesh II, Rozen-Zvi B, Kalechman Y, Gafter U, Sredni B (2014). AS101 prevents diabetic nephropathy progression and mesangial cell dysfunction: regulation of the AKT downstream pathway. PLoS One.

[4] Mason RM, Wahab NA (2003). Extracellular matrix metabolism in diabetic nephropathy. J Am Soc Nephrol.

[5] Xu F, Wang Y, Cui W (2014). Resveratrol prevention of diabetic nephropathy is associated with the suppression of renal inflammation and mesangial cell proliferation: possible roles of Akt/NF-kappaB pathway. Int J Endocrinol.

[6] Loganathan TS, Sulaiman SA, Abdul Murad NA, Shah SA, Abdul Gafor AH, Jamal R, Abdullah N (2020). Interactions among non-coding RNAs in diabetic nephropathy. Front Pharmacol.

[7] Long J, Badal SS, Ye Z, Wang Y, Ayanga BA, Galvan DL, Green NH, Chang BH, Overbeek PA, Danesh FR (2016). Long noncoding RNA Tug1 regulates mitochondrial bioenergetics in diabetic nephropathy. J Clin Invest.

[8] Li SY, Susztak K (2016). The long noncoding RNA Tug1 connects metabolic changes with kidney disease in podocytes. J Clin Invest.

[9] Kato M, Wang M, Chen Z, Bhatt K, Oh HJ, Lanting L, Deshpande S, Jia Y, Lai JYC, O’Connor CL, Wu YF, Hodgin JB, Nelson RG, Bitzer M, Natarajan R (2016). An endoplasmic reticulum stress-regulated lncRNA hosting a microRNA megacluster induces early features of diabetic nephropathy. Nat Commun.

[10] Wang S, Chen X, Wang M, Yao D, Chen T, Yan Q, Lu W (2018). Long non-coding RNA CYP4B1-PS1-001 inhibits proliferation and fibrosis in diabetic nephropathy by interacting with Nucleolin. Cell Physiol Biochem.

[11] Zhang P, Sun Y, Peng R, Chen W, Fu X, Zhang L, Peng H, Zhang Z (2019). Long non-coding RNA Rpph1 promotes inflammation and proliferation of mesangial cells in diabetic nephropathy via an interaction with Gal-3. Cell Death Dis.

[12] Jiang L, Wang R, Fang L, Ge X, Chen L, Zhou M, Zhou Y, Xiong W, Hu Y, Tang X, Li G, Li Z (2019). HCP5 is a SMAD3-responsive long non-coding RNA that promotes lung adenocarcinoma metastasis via miR-203/SNAI axis. Theranostics..

[13] Liang L, Xu J, Wang M, Xu G, Zhang N, Wang G, Zhao Y (2018). LncRNA HCP5 promotes follicular thyroid carcinoma progression via miRNAs sponge. Cell Death Dis.

[14] Li N, Cui Y, Yin M, Liu F (2019). Screening potential prognostic biomarkers of long non-coding RNAs for predicting the risk of chronic kidney disease. Braz J Med Biol Res.

[15] Lu CC, Ma KL, Ruan XZ, Liu BC (2017). The emerging roles of microparticles in diabetic nephropathy. Int J Biol Sci.

[16] Yang J, Shen Y, Yang X, Long Y, Chen S, Lin X, Dong R, Yuan J (2019). Silencing of long noncoding RNA XIST protects against renal interstitial fibrosis in diabetic nephropathy via microRNA-93-5p-mediated inhibition of CDKN1A. Am J Physiol Renal Physiol.

[17] Su L, Deng Z, Leng F. The Mammalian High Mobility Group Protein AT-Hook 2 (HMGA2): Biochemical and Biophysical Properties, and Its Association with Adipogenesis. Int J Mol Sci. 2020;21(10):3710. 10.3390/ijms21103710. 10.3390/ijms21103710 PMC7279267 32466162

[18] Wang Y, Le Y, Xue JY, Zheng ZJ, Xue YM (2016). Let-7d miRNA prevents TGF-beta1-induced EMT and renal fibrogenesis through regulation of HMGA2 expression. Biochem Biophys Res Commun.

[19] Xu JL, Gan XX, Ni J, Shao DC, Shen Y, Miao NJ, Xu D, Zhou L, Zhang W, Lu LM (2018). SND p102 promotes extracellular matrix accumulation and cell proliferation in rat glomerular mesangial cells via the AT1R/ERK/Smad3 pathway. Acta Pharmacol Sin.

[20] Teng H, Wang P, Xue Y, Liu X, Ma J, Cai H, Xi Z, Li Z, Liu Y (2016). Role of HCP5-miR-139-RUNX1 feedback loop in regulating malignant behavior of glioma cells. Mol Ther.

[21] Ulbing M, Kirsch AH, Leber B, Lemesch S, Münzker J, Schweighofer N, Hofer D, Trummer O, Rosenkranz AR, Müller H, Eller K, Stadlbauer V, Obermayer-Pietsch B (2017). MicroRNAs 223-3p and 93-5p in patients with chronic kidney disease before and after renal transplantation. Bone..

[22] Wang T, Zhu H, Yang S, Fei X. Let7a5p may participate in the pathogenesis of diabetic nephropathy through targeting HMGA2. Mol Med Rep. 2019;19(5):4229–37. 10.3892/mmr.2019.10057. 10.3892/mmr.2019.10057 PMC6471493 30896854

[23] Zhu Y, Xu J, Liang W, et al. miR-98-5p Alleviated Epithelial-to-Mesenchymal Transition and Renal Fibrosis via Targeting Hmga2 in Diabetic Nephropathy. Int J Endocrinol. 2019;2019:4946181. 10.1155/2019/4946181. 10.1155/2019/4946181 PMC6925681 31885559

[24] Xiang H, Xue W, Wu X, Zheng J, Ding C, Li Y, Dou M (2019). FOXP1 inhibits high glucose-induced ECM accumulation and oxidative stress in mesangial cells. Chem Biol Interact.

[25] Huang S, Xu Y, Ge X, Xu B, Peng W, Jiang X, Shen L, Xia L (2019). Long noncoding RNA NEAT1 accelerates the proliferation and fibrosis in diabetic nephropathy through activating Akt/mTOR signaling pathway. J Cell Physiol.

